# Orthologs, turn-over, and remolding of tRNAs in primates and fruit flies

**DOI:** 10.1186/s12864-016-2927-4

**Published:** 2016-08-11

**Authors:** Cristian A. Velandia-Huerto, Sarah J. Berkemer, Anne Hoffmann, Nancy Retzlaff, Liliana C. Romero Marroquín, Maribel Hernández-Rosales, Peter F. Stadler, Clara I. Bermúdez-Santana

**Affiliations:** 1 Biology Department, Universidad Nacional de Colombia, Carrera 45 # 26-85, Edif. Uriel Gutiérrez, Bogotá, D.C Colombia; 2 Max Planck Institute for Mathematics in the Sciences, Inselstraße 22, Leipzig, D-04103 Germany; 3 Bioinformatics Group, Department of Computer Science, and Interdisciplinary Center for Bioinformatics, Universität Leipzig, Härtelstraße 16–18D-04107, Leipzig, Germany; 4 CONACYT – Instituto de Matemáticas, UNAM Juriquilla, Av. Juriquilla #3001, Santiago de Querétaro, MX-76230 QRO México; 5 Fraunhofer Institut for Cell Therapy and Immunology, Perlickstraße 1, Leipzig, D-04103 Germany; 6 Department of Theoretical Chemistry, University of Vienna, Währinger Straße 17, Vienna, A-1090 Austria; 7 Center for non-coding RNA in Technology and Health, Grønegårdsvej 3, Frederiksberg C, DK-1870 Denmark; 8 Santa Fe Institute, 1399 Hyde Park Rd., Santa Fe, NM87501 USA

**Keywords:** Concerted evolution, tRNA remolding, Synteny, Orthology

## Abstract

**Background:**

Transfer RNAs (tRNAs) are ubiquitous in all living organism. They implement the genetic code so that most genomes contain distinct tRNAs for almost all 61 codons. They behave similar to mobile elements and proliferate in genomes spawning both local and non-local copies. Most tRNA families are therefore typically present as multicopy genes. The members of the individual tRNA families evolve under concerted or rapid birth-death evolution, so that paralogous copies maintain almost identical sequences over long evolutionary time-scales. To a good approximation these are functionally equivalent. Individual tRNA copies thus are evolutionary unstable and easily turn into pseudogenes and disappear. This leads to a rapid turnover of tRNAs and often large differences in the tRNA complements of closely related species. Since tRNA paralogs are not distinguished by sequence, common methods cannot not be used to establish orthology between tRNA genes.

**Results:**

In this contribution we introduce a general framework to distinguish orthologs and paralogs in gene families that are subject to concerted evolution. It is based on the use of uniquely aligned adjacent sequence elements as anchors to establish syntenic conservation of sequence intervals. In practice, anchors and intervals can be extracted from genome-wide multiple sequence alignments. Syntenic clusters of concertedly evolving genes of different families can then be subdivided by list alignments, leading to usually small clusters of candidate co-orthologs. On the basis of recent advances in phylogenetic combinatorics, these candidate clusters can be further processed by cograph editing to recover their duplication histories. We developed a workflow that can be conceptualized as stepwise refinement of a graph of homologous genes. We apply this analysis strategy with different types of synteny anchors to investigate the evolution of tRNAs in primates and fruit flies. We identified a large number of tRNA remolding events concentrated at the tips of the phylogeny. With one notable exception all phylogenetically old tRNA remoldings do not change the isoacceptor class.

**Conclusions:**

Gene families evolving under concerted evolution are not amenable to classical phylogenetic analyses since paralogs maintain identical, species-specific sequences, precluding the estimation of correct gene trees from sequence differences. This leaves conservation of syntenic arrangements with respect to “anchor elements” that are not subject to concerted evolution as the only viable source of phylogenetic information. We have demonstrated here that a purely synteny-based analysis of tRNA gene histories is indeed feasible. Although the choice of synteny anchors influences the resolution in particular when tight gene clusters are present, and the quality of sequence alignments, genome assemblies, and genome rearrangements limits the scope of the analysis, largely coherent results can be obtained for tRNAs. In particular, we conclude that a large fraction of the tRNAs are recent copies. This proliferation is compensated by rapid pseudogenization as exemplified by many very recent alloacceptor remoldings.

**Electronic supplementary material:**

The online version of this article (doi:10.1186/s12864-016-2927-4) contains supplementary material, which is available to authorized users.

## Background

The reconstruction of detailed evolutionary histories of gene families is a prerequisite for dating and understanding innovations, see e.g. [1, 2]. It plays an important role in particular in the emerging field of forward genomics [3]. Of particular importance is the distinction between orthologs, i.e., gene pairs that originated from a speciation event, and paralogs, which arose by gene duplication [4]. Orthology detection is usually based on evolutionary distances that are estimated from sequence similarities, and proceeds either directly using a “reciprocal best match” approach [5] or indirectly by computing a gene phylogeny and its reconciliation with the species tree, see e.g. [6–8] for reviews. Both approaches make the assumption that distinct genes evolve essentially independently, so that their evolutionary distance is strongly correlated with and thus can be inferred from sequence similarity. This assumption is violated, however, in many cases, which include ribosomal RNA and other RNA gene families as well as some protein-coding gene families such as histones [9].

### Concerted evolution

Most transfer RNAs are multi-copy genes that are dispersed throughout the genome. Paralogous tRNAs with the same anti-codon nevertheless maintain (nearly) identical sequences over extreme evolutionary time-scales. This effect is known as *concerted evolution* [10]. It was shown already in the 1980s that intergenic conversion is an important contributing factor [11]. Ectopic gene conversion involves the unidirectional transfer of sequence genetic material from a “donor” sequence to a highly similar “acceptor” [12, 13]. Due to the extremely low rates of sequence evolution in tRNAs, gene conversion events are frequent enough for the information transfer to be effectively bidirectional. Hence the entire set of nearly identical paralogs is kept coherent throughout evolution. Gene conversion is also responsible for preventing the divergence of the individual copies of the ribosomal RNA cistron [14] and histone genes [15]. In many case genes evolving under concerted evolution are arranged in genomically localized clusters.

### Evolution of transfer RNAs

Transfer RNAs, like many other classes of small RNA genes, such as snoRNAs [16], behave as mobile genetic elements. In fact, the universal class of SINE elements derives from tRNAs [17]. As a consequence, the tRNA repertoire can change very rapidly even between closely related genomes [18, 19]. In addition, tRNAs appear to proliferate by tandem duplications, leading to the formation of tRNA clusters. Simulation studies [12] show that duplicate genes will not remain subject to concerted evolution forever, but will escape with a roughly exponentially distributed waiting time and start to accumulate mutations [12]. In the case of tRNAs this typically leads to pseudogenization. One therefore observes a sometimes rapid net turnover of tRNA genes at individual loci [18, 20–22]. Turnover can be estimated quite accurately by simply comparing gene copy numbers between species when gain and loss events are rare as in the case of microRNAs [23]. As we have seen in previous work [18], however, the fraction of conserved tRNA loci quickly decreases with phylogenetic distance, so that similar tRNA numbers among different mammalian families are the consequence of compensation between large numbers of gain and loss events.

In addition to gain and loss of entire tRNA genes, mutations in the anticodon loops may change the identity of the tRNA. This process is known as *tRNA remolding* [24]. The modified anticodon usually corresponds to the same aminoacid (isoacceptor remolding), however, in mitochondrial genomes also alloacceptor remoldings, i.e., a change in the addressed aminoacid, is observed with surprising frequency [25, 26]. The nuclear tRNAs of eukaryotes, in contrast, are largely restricted to isoacceptor remoldings [22, 27], presumably because proper loading of a tRNA depends on a complex system of identifying elements that may even be disjoint from the anticodon sequence [28]. Surprisingly, even isoacceptor remoldings are rare in Archaea and Eubacteria [27]. Like the estimation of tRNA gain and loss, a quantitative investigation of tRNA remolding events also hinges on the correct identification of orthology.

### Computational approaches

In this contribution we introduce a general workflow to accurately estimate orthology for partly clustered multigene families that evolve under concerted evolution. An extensive body of literature describes algorithms and software to reconstruct gene cluster histories, see [29–34]. They require sequences, sequence similarities, or gene trees as input and explicitly or implicitly rely on the possibility to infer evolutionary distances from sequence information. In the case of concerted evolution, however, this is not possible because the sequences of the family members within each species are essentially identical [9]. Even paralogs that have escaped concerted evolution carry no informative signal about the time before their escape.

Consequently, a completely different approach is required. The most reliable alternative source of information is syntenic conservation, i.e., preservation of *relative* positions within the genomic DNA sequence. It was exploited in [20, 22] to devise a strategy in which the query tRNA is embedded in intervals of flanking sequences whose size is increased until a unique blast match in the target genome is found. In this manner, an approximation to orthology is obtained. By the time the uniqueness condition is satisfied, however, intervals may extend across entire tRNA clusters, calling for methods to further refine the orthology assignments.

Here we investigate the idea of synteny-based orthology identification in a more systematic manner. On the one hand, we ask whether pre-computed genome-wide alignments can efficiently be used for this purpose. On the other hand, we show that it is possible to at least approximate also the history of gene clusters that cannot be resolved further on the basis of sequence-based synteny data. To this end we combine an alignment-like approximation to multi-species synteny with recent advances in phylogenetic combinatorics [35, 36] that relate orthology with cographs. In the following section we discuss the individual components of our workflow in detail. As an application we then revisit the evolution of tRNAs in primates, as an example for a phylogenetically very narrow range, and in fruit flies, as an example for a phylogenetically already very diverse system.

## Theory

Our approach consists of three conceptual steps: first synteny is used to narrow down candidate co-orthologs to local tRNA clusters. In the second step each of the initial candidate sets is partitioned further based on sequence similarity on preserved relative order, resulting in an estimated co-orthology relation. These are further refined and corrected using the fact that orthology relations must have cograph structures. In the remainder of this section we describe the individual steps formally and discuss possible future improvements.

### From synteny to candidate orthologs

We consider a set *Σ* of species or genomes. Each genome *a*∈*Σ* comprises a discrete set of loci. Genomic coordinates establish an order relation ≺ among loci. Since genetic elements have an intrinsic reading direction the order ≺ is either the same or the inverse of the coordinate system. We write $\bar {u}^{a}$ for the reverse complement of locus *u*^*a*^ on genome *a*. Note that *u*^*a*^≺*v*^*a*^ is equivalent to $\bar v^{a}\prec \bar {u}^{a}$. Since the reverse complement of a locus is also a valid locus we arbitrarily choose the orientation.

For a subset of loci we assume that they evolve independently by vertical inheritance and are not subject to duplication in the set of species under consideration. We say that two tRNAs *t*^*a*^ and *t*^*b*^ in genomes *a* and *b*, respectively, are 1:1 orthologs, if *t*^*a*^ is the only ortholog in genome *a* of *t*^*b*^ in genome *b*, and *vice versa*. Therefore we know (or can compute) the 1:1 orthologs of *p*^*a*^ in a set of species *Σ*_*p*_⊆*Σ*. We will refer to such a set of orthologous loci *p*={*p*^*a*^|*a*∈*Σ*_*p*_} as an *anchor*. An anchor *p* may connect all or only a subset *Σ*_*p*_⊆*Σ*. The orthologs within an anchor are defined to be oriented in the same reading direction. Therefore, if *p* and *q* are anchors with *p*^*a*^≺*q*^*a*^ then *p*^*b*^≺*q*^*b*^ for all *a*,*b*∈*Σ*_*p*_∩*Σ*_*q*_. That is, we assume that anchors preserve synteny including relative reading direction in the set of genomes of interest. We can therefore write *p*≺*q*.

Now we consider a set *T* of loci of interest; in our case tRNAs. None of the *t*^*a*^∈*T* gives rise to an anchor, i.e., we assume that the multiple, nearly identical sequences are present in the genome. We make two basic, simplifying assumptions:

- There are anchors *p* and *q* such that *p*^*a*^≺*t*^*a*^≺*q*^*a*^.
- A pair of anchors can be chosen such that the relative order such that the order of homologous loci in preserved between *p* and *q*.

Both assumptions are approximations to reality. Condition (S1) stipulates that the locus *t*^*a*^ of interest is not too close to the end of contig, scaffold, or chromosome. It will be violated essentially by incomplete data and flaws in genome assemblies. Condition (S2) is a more severe restriction. It allows only unduplicated vertical inheritance and tandem duplications of individual loci. It explicitly rules out genome arrangement between anchors sufficiently close to the locus of interest and also neglects tandem duplications affecting more than a single gene. In essence it forces us to treat a multi-locus tandem duplication as if it was a combination of unduplicated vertical inheritance combined with the insertion of the second copy of tandem pair. It could be replaced in future work by the weaker condition that genomic evolution by tandem duplication, and regarrangements is confined to regions delimited by the anchors.

The exact nature of the anchors is irrelevant for the workflow. In a very conservative approach, known sets of orthologous protein-coding mRNAs are used. If a more fine-grained resolution is desired, one can use e.g. blocks of genome-wide multiple sequence alignment (see Methods).

#### Tight anchors

Condition (S1) allows us to obtain initial candidates for orthology assignments. We assume that they have a set of homologous elements *T*_*a*_ for each genome *a*∈*Σ*. If *p* and *q* are anchors with *p*^*a*^≺*t*^*a*^≺*q*^*a*^ then any *t*^′^∈*T*_*b*_ with *t*^′^≺*p*^*b*^ or *q*_*b*_≺*t*^′^ cannot be co-ortholog of *t*^*a*^ in genome *b*.

**Fig. 1:**
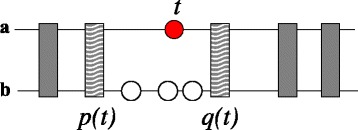
Tight anchors for *t* into species *b*. Possible anchors are indicated by the *grey boxes*. The tight anchors are the anchors closest to *t* (marked in *lighter grey*) that connect species *a* and *b*. By synteny, the only possible orthologs of *t* are the three loci indicated by *white circles*

The practical difficulty is that in general we might not have anchors that cover all species of interest but only a subset of them. For any “query” locus *t*^*a*^ and any species *b*∈*Σ*, *b*≠*a* we therefore define a *pair of tight anchor fort*^*a*^* into b* as a pair of anchors *p*_*b*_(*t*^*a*^):={*p*^*a*^,*p*^*b*^} and *q*_*b*_(*t*^*a*^)={*q*^*a*^,*q*^*b*^} such that (i) *p*^*a*^≺*t*^*a*^≺*q*^*a*^ and (ii) the pair (*p*_*b*_(*t*^*a*^),*q*_*b*_(*t*^*a*^)) is minimal in the sense that there is no further anchor *u*=(*u*^*a*^,*u*^*b*^) with *p*^*a*^≺*u*^*a*^≺*t*^*a*^ or *t*^*a*^≺*u*^*a*^≺*q*^*a*^, Fig. 1.

Under our assumption (S1), there is a unique pair of tight anchors of *t*^*a*^ into *b* for every *b*∈*Σ*. In practice, however, there may be exceptions: in the case of genome arrangement or a fragmented genome assembly the anchor points *p*_*b*_(*t*^*a*^) and *q*_*b*_(*t*^*a*^) may be located on very far apart or even on different chromosomes, contigs, or scaffold. We refer to the Methods section for the handling of such exceptions.

Condition (S2), or even a much weaker locality assumption, implies that only the homologs *t*^′^∈*T*_*b*_ enclosed by the pair of tight anchors for *t*^*a*^ are possible co-orthologs of *t*^*a*^ in genome *b*.

#### Candidate graph

**Fig. 2:**
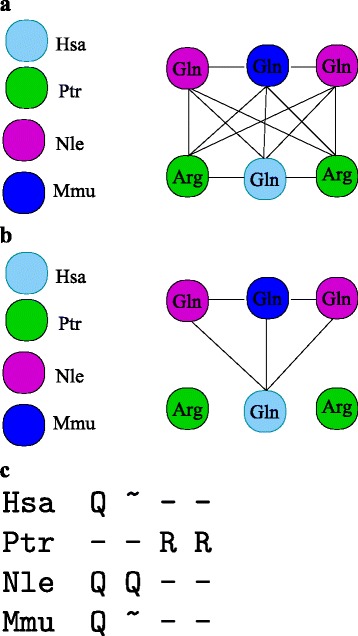
Stepwise refinement of the candidate graph *Γ*_*c*_. **a** The graph *Γ*_*c*_ represents the possible orthology assignments among tRNA loci derived from the synteny anchors. Only genes from different species can be orthologs, hence no edges connect loci in the same species. **b** Based on sequence similarity edges are removed between tRNAs from different isoacceptor families. **c** A modified Needleman-Wunsch alignment algorithm is used to identify order-preserving subgroups. This step admits local tandem duplications but not duplications of larger subclusters

From the sets of homologous loci *T*^*a*^ and a collection of anchors on *Σ* we construct the *candidate graphΓ*_*c*_ as follows. The vertices *Γ*_*c*_ are the annotated homologs, i.e., $T=\bigcup _{a\in \Sigma } T_{a}$. An edge between *t*^*a*^∈*T*_*a*_ and *t*^*b*^∈*T*_*b*_ is inserted if *p*_*b*_(*t*^*a*^)≺*t*^*b*^≺*q*_*b*_(*t*^*a*^), i.e., if *t*^*b*^ is located between the pair of tight anchors from *t*^*a*^ into *b*. In order to accomodate some local inversions and/or assembly errors one might want to relax this definition and to draw an edge between *t*^*a*^ and every locus *t*∈*T*_*b*_ so that *p*_*b*_(*t*^*a*^)≺*t*≺*q*_*b*_(*t*^*a*^) or $p_{b}(t^{a})\prec \bar t\prec q_{b}(t^{a})$. By construction, the true orthology relation is a sub-graph of *Γ*_*c*_, see Fig. 2a. Its nodes are the tRNAs and there is an edge between two tRNAs if they are possibly orthologuous, thus if they are flanked by the same tight anchors and belong to distinct species.

The graph *Γ*_*c*_ is not sufficient to completely solve the orthology problem because in general two tRNA loci ${t^{a}_{i}}$ and ${t^{a}_{j}}$ will not be separated by anchors. The available anchors in fact may enclose entire tRNA clusters, see Fig. 1. For tRNAs, however, we can clearly distinguish subgroups by sequence similarity. In particular, tRNAs of different isoacceptor families (i.e., those that are loaded with different aminoacids) and within these, most subgroups with distinct anticodons, exhibit clearly separate sequences. We therefore can prune the edge set of *Γ*_*c*_ by removing all edges that connect tRNA loci with clearly distinct sequences. We therefore require that the genetic distance satisfies $d_{G}({t^{a}_{i}},{t^{b}_{j}})<\varepsilon $ for all edges of the pruned candidate graph, which we denote by *Γ*_*a*_, see Fig. 2b. The threshold *ε* is chosen as an upper bound on divergence of genes in phylogenetic range of interest (see Methods for details).

### Order preservation within clusters

Assumption (S2), stipulates that co-orthologous loci preserve relative order. In the context of tRNA clusters, this amounts to the assumption that tRNAs within a gene cluster proliferate by means of single gene tandem duplications or by retroposition-like insertions.

The relationship between clustered tRNAs in two species corresponds to a generalized version of an alignment problem. In order to see this, we consider each tRNA cluster as an ordered list of tRNAs and tRNA pseudogenes ${t^{a}_{i}}$ and ${t^{b}_{j}}$ in the two genomes *a* and *b*. For the sake of the argument let us first neglect gene duplications and consider insertion, deletion, and remolding only. In this case the correspondences between orthologous loci form an order-preserving matching in the induced subgraph of *Γ*_*a*_ restricted to every pair of species. This amounts to an alignment of the tRNA loci in *T*_*a*_ with those in *T*_*b*_ with alignment edges allowed only between loci that are connected by an edge in *Γ*_*a*_.

#### Modified Needleman-Wunsch alignment

In order to account for local, i.e., order-preserving duplications we can extend the alignment model in a simple manner. In the usual setting of matchings, one locus ${t^{a}_{i}}$ can match at most a single locus ${t^{b}_{j}}$. Otherwise one of ${t^{a}_{i}}$ and ${t^{b}_{j}}$ is deleted. This is called a 1:1 alignment. In the simplest extension also 1:2 and 2:1 matches are allowed, i.e, two positions $({t^{a}_{i}}, t^{a}_{i+1})$ may collectively match a single position ${t^{b}_{j}}$, or *vice versa*. More generally, *p*:*q* matches may be considered. Such extensions to one-to-many or many-to-many matches lead to quite simple modification of the Needleman-Wunsch [37] algorithm (see Methods for details). Such extensions have been proposed and applied to natural language data, see e.g. [38, 39] and a means of extracting co-linear clusters of phylogenetic footprints [40].

As stated above, condition (S2) is a restrictive approximation that rules out tandem duplications of subclusters larger than a single locus as well as any local genome rearrangements. More inclusive assumption could be made instead. The full duplication-loss alignment problem that allows copying of subclusters of arbitrary size is APX hard [41], but a practicable dynamic programming heuristic is available [42]. Recently, it was extended further in OrthoAlign to include also genome rearrangements [43]. In principle these approaches could be substituted into our workflow. We are content here with the simpler list alignment method, Fig. 2c, despite its shortcomings because it allows us to avoid the problem of estimating weight parameters for complex duplications and rearrangments operations. As an alternative to alignment-like approaches for disentangling the history of individual loci it may also be fruitful to consider generalizations of gene order methods, see e.g. MLGO [44–47], albeit at least in the data we considered here duplications and losses are by far the dominating events.

#### Linear coordinate interpolation

An even stricter alternative to the list alignments is to assume that not only the relative order but also the relative distances of gene are preserved. Given two anchors *p* and *q* and an annotated tRNA position *t*^*a*^, one can then estimate the position of its putative ortholog *t*^∗^ by linear interpolation:

1 $$  t^{*} = p^{b} + \frac{q^{b}-p^{b}+1}{q^{a}-p^{a}+1} t^{a}  $$

**Fig. 3:**
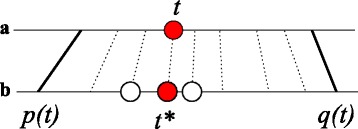
Determining orthologs by linear coordinate transformations

The fraction in Eq.(1) is simply the ratio of the sequence lengths between the corresponding anchor points in the two genomes *b* and *a*, resp., see Fig. 3. This simple estimate was used e.g. in [48, 49] in the context of phylogenetic footprinting. The tRNA in *b* closest to the predicted position *t*^∗^ is the best estimate for a 1:1 ortholog. It is important to note that the linear interpolation method can detect only 1:1 orthologs and will fail for co-orthologs arising from gene duplications.

#### Estimated orthology graph

**Fig. 4:**
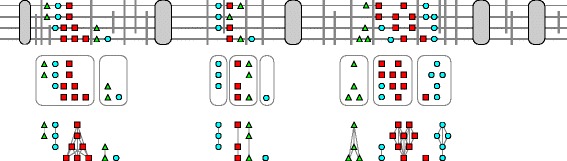
Scheme of step-wise orthology identification. Top genomic organization of tRNAs (*colored symbols*). “Secure anchors” such as known orthologous proteins are shown as *gray ovals*. Sequence-unique alignment blocks are indicated as *thick dark-gray lines*. These anchors subdivide the genome into syntenic clusters forming the connected components of the graph of candidates *Γ*_*c*_, here shown for blocks of a genome-wide alignment as delimiters. Each cluster forms a connected component of *Γ*_*c*_. Pairwise generalized list alignments leads to an estimate of the co-orthology relation for each group of homologous tRNAs. Each of these estimated graphs is then corrected to the nearest cograph

The alignment edges predicted by the pairwise generalized alignment algorithm serve our best estimates for the orthology relation. For 1:2 duplications an edge is inserted from the “original” to both “copies”; in the more general case of *p*:*q* duplications, we accept all edges of the complete bipartite graph corresponding to the *p*:*q* duplication. Superimposing all pairwise alignments yields the *estimated orthology graphΓ*_*o*_, conceptually shown in the bottom row of Fig. 4. It contains only edges between tRNAs that can be orthologs according to their sequence similarity, and all connected components of *Γ*_*o*_ are order preserving since their edges result from the order-preserving alignment step, see Fig. 4. By construction *Γ*_*o*_ is a spanning subgraph of *Γ*_*a*_, which in turn is a spanning subgraph of the initial candidate graph *Γ*_*c*_. In general, *Γ*_*o*_ will consist of many small connected components, each comprising members of a single tRNA family that locally has expanded and contracted by duplication and loss events.

### Cographs and orthology

Recent results in phylogenetic combinatorics [35, 36, 50–53] show that orthology relations are cographs. There are many equivalent characterizations for this well-studied class. In particular, *G* is a cograph if it does not contain a *P*_4_, a path on 4 vertices, as an induced subgraph [54]. In particular, complete graphs are cographs. A cograph is associated with a unique cotree, which corresponds to the (not necessarily fully resolved) gene tree with labels at the interior vertices that identify speciation and duplication events, respectively [35, 36].

We expect that *Γ*_*o*_ is already a very good approximation to tRNA orthology. Various sources of noise, however, will introduce violations of the cograph structure. Therefore, the orthology estimates can be improved further by editing *Γ*_*o*_ to the nearest cograph. This amounts to inserting and deleting the minimal number of edges so that all *P*_4_s are destroyed. Although the cograph editing problem is NP hard [55], this is not a practical problem here. It is not difficult to see that the connected components *C*_*i*_ of *Γ*_*o*_ can be edited independently of each other [36]. Empirically, we observe that most connected components of *Γ*_*o*_ are complete graphs and this already correct cographs.

From the final, corrected orthology estimates $\hat {C}_{i}$ it is now straightforward to infer the evolutionary events. The cographs $\hat {C}_{i}$ themselves provide direct information on the tandem duplication events. To this end it suffices to convert the $\hat {C}_{i}$ into its equivalent cotree [54], from which the duplication events can be directly read off. Deletion events as well as gain events in which a particular locus was settled are obtained by mapping each of the $\hat {C}_{i}$ to the species tree. We use Dollo parsimony [56] approach to derive the numbers of gain, losses, and duplications from the co-ortholog groups. Duplication events identified from the cographs $\hat {C}_{i}$ are counted separately from gains.

## Results and discussion

### Evolution of primate tRNAs

Starting from the primate multiz alignments [57] we obtained 1665 connected components of *Γ*_*c*_, including 961 singletons. In 168 connected components, tRNAs of only a single species were found. 536 connected components formed non-trivial graphs showing the orthology relation between tRNAs in distinct species. Almost all of the connected components of *Γ*_*o*_ were already cographs. Only 3 of the 536 graphs had a non-cograph structure. This appears to be related to pseudogenization of part of the cluster, which causes some of the pairwise distances of the pseudogenized tRNAs to drop below the threshold value for orthology assignment.

**Fig. 5:**
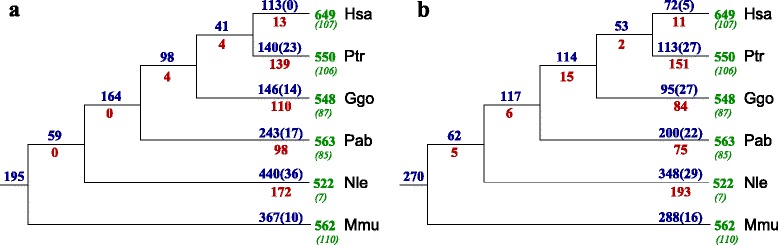
**a** Gain, loss, and duplications of tRNAs in primates computed from the most fine-grained synteny definition based on individual MSA blocks and **b** by joining adjacent blocks as described in the text. Gain and duplication events were assigned to the edge leading to the last common ancestor of all observed co-orthologs, except for groups that contained only a macaque and a human or a chimpanzee tRNA; in these cases we assigned two lineage specific gains. *Green numbers* refer to the total number of tRNAs detected by tRNAscan-SE; *green numbers in parentheses* count the pseudogenes found in the set of all tRNAs. *Blue numbers* refer to the total gain, i.e., the sum of event seeding new connected components and duplication events with a connected component. The number of identified local duplication events is given in *parentheses in blue*. The *red numbers* indicate the loss events on the corresponding branch. Species abbreviations: human, *Homo sapiens*: Hsa; chimapanzee, *Pan troglodytes*: Ptr; gorilla, *Gorilla gorilla*: Ggo; orangutan, *Pongo abelii*: Pab; gibbon, *Nomascus leucogenys*: Nle; rhesus macaque, *Macaca mulatta*: Mmu

The connected components based on the multiz MSA (multiple sequence alignment) blocks are typically small and show very few tandem duplications. This may be caused by the choice of one particular copy of duplicated sequence flanking a tRNA in the multiz pipeline. The corresponding gain and loss events are mapped to the primate phylogeny in Fig. 5. To investigate this effect we therefore joined connected components of the multiz-based *Γ*_*c*_ that share boundary MSA blocks. This reduced the number of synteny regions by about a third to 1079 connected components and about halved the number of singleton from 961 to 482. Still, we found 64 components comprising tRNAs of only a single species. Of 533 non-trivial connected components only 2 did not have cograph structure.

The main effect joining adjacent synteny groups, i.e., considering larger syntenic groups in the initial step, is that events are assigned to evolutionary more ancient events. This is a consequence of reconstructing larger clusters as the ancestral state, so that more deletions from these clusters are inferred instead of evolutionary more recent events of seeding novel clusters.

**Fig. 6:**
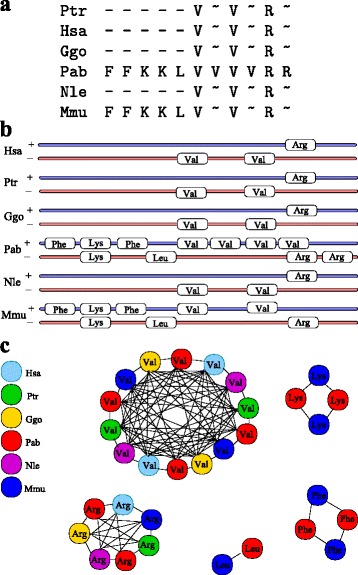
A more complex tRNA cluster in primates (see Additional file 5 for coordinates. Panel **a** summarizes the situation as list alignment. For simplicity, tRNAs from both strands are included. Except in rhesus and orangutan the first part of cluster the has been cluster. Panel **b** shows a more detailed, strand-specific genomic map. It highlights the reversal of the orientation of a tRNA Arg (R) in rhesus and the two copies of tRNA Lys (K) on opposite strands. Panel **c** shows the graph corresponding to the cluster. Edges indicate that the tRNA sequences sufficiently similar by be possible orthologs. Different species are distinguished by colors. The tRNAs isoacceptor classes are indicated by their 1-letter codes: Phe (F), Lys (K), Leu (L), Val (V), Arg (R)

An example of a more complex cluster is shown in Fig. 6. Here, part (a) shows the list alignment of the whole cluster for all species. It can be seen that several duplication events occured for *P. abelii*. Looking closer at this cluster, one can see that the detected tRNAs are located on different strands, as shown in part (b) of the figure. Additionally, it can be seen that for *P. abelii* and *M. mulatta* orthologous tRNAs are located on a different strand than in the other species. Thus, the tRNAs switched strand but kept their ordering. Part (c) shows the corresponding graphs where the nodes are the tRNAs and two tRNAs are connected if they sequences are highly similar and they are in a syntenic relation. Here, no difference is made for tRNAs that are on different strands as their synteny still holds. We do not have a good explanation for the discrepancy between the large clusters and strand switching in *P. abelii* and *M. mulatta* whereas *N. leucogenys* retained a much smaller cluster. Conceivably it is an artefact of the genome assembly and/or the genome wide alignment.

Of the cographs, 327 were cliques and thus did not contain duplication events. The remaining 206 include duplication events that increased the total number of tRNAs by 66. In addition, 60 duplications were detected in the connected components containing only tRNAs of the same species.

In summary, we observe that between about a third and a half of the tRNAs in extant primate genomes have been syntenically conserved since the last common ancestor of human and macaque. The seeding of new tRNA locations, on the other hand, is clearly an ongoing process. A surprisingly large number of loci, is gained and lost in a lineage-specific manner. This effect can be attributed to the rapid formation and erasure of pseudogenized copies. Errors in the genome assembly and the genome-wide sequence alignments will lead to false negatives in the synteny assessment and thus to unrecognized orthologies.

**Fig. 7:**
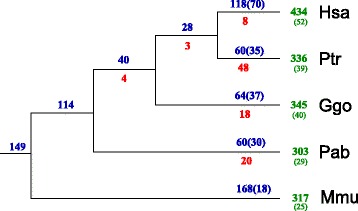
Gain, loss and duplications of tRNAs in primates computed based on protein-anchored clusters and the linear interpolation method. *N. leucogenys* was not included in this part of the analysis

Using the much sparser orthologous protein anchors only 231 genomic clusters of tRNAs, of which 166 are nontrivial, were identified. The smaller is in part due to a larger number of tRNAs per cluster and in part because in particular many tRNA pseudogenes were not located in a traceable cluster. With only 7 exceptions the protein-anchored clusters are cographs. The exceptional cases were easily converted to cographs by adding a small number edges, i.e., as cograph completions [58]. Missing edges apparently correspond to pairs of likely pseudogenized tRNAs with a sequence distance exceeding the 10 % threshold. The number of evolutionary events derived from the protein-anchored clusters is significantly smaller than with more fine-grained synteny anchors, Fig. 7.

The number of 4957 ortholog edges in the protein anchored data approach is higher than in the MSA based approach since the average number of tRNAs per cluster is higher for the protein anchored clusters. Of the 1986 orthology edges identified by the MSA approach, 1653 (83 %) are recovered based on the protein anchors. Much of remainder is explained by the exclusion of tRNAs that could not be associated with orthologous flanking proteins. For details we refer to Additional file 1.

### Evolution of tRNAs in fruit flies

Drosophilids cover evolutionary distances comparable to the entire vertebrate phylum [59]. Nevertheless the synteny-based method of ortholog identification remains applicable since the much smaller genomes still provide a sufficient density of anchors with unique sequence.

Based on the multiz alignments provided through the UCSC genome browser we identified 1889 connected components including 1235 singletons. 375 connected components contained tRNAs of just one species. The remaining 280 connected components were graphs showing the orthology relations between tRNAs of distinct species. Out of these, 275 graphs have a cograph structure and in only 5 cases the graph structure had to be edited to get the closest possible cograph structure. Analogously, for the primate case, clusters were then joined such that two clusters sharing the same border became one cluster. This reduced the number of connected components by about 40 % to 1042, of which 722 did not have any edges. 602 of these graphs were singleton tRNAs and in the remaining 110 only tRNAs of the same species were found. All the 320 non-trivial graphs were cographs. Out of these, 190 cographs were cliques. In the remaining 130 graphs, 205 duplicated tRNAs could be detected. Additionally, 349 duplications were detected in the graphs containing tRNAs of the same species.

**Fig. 8:**
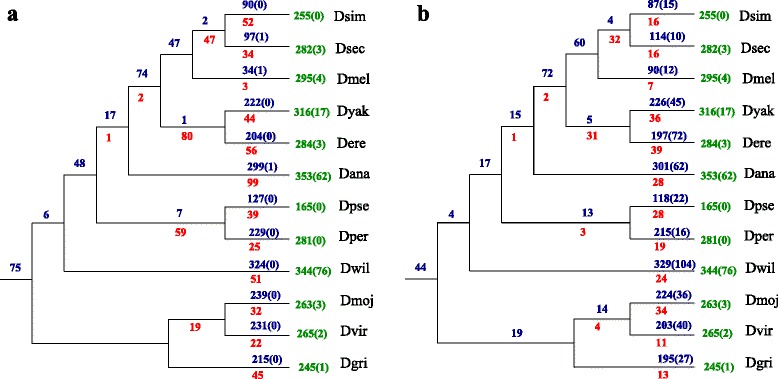
Gains **a** and losses **b** of tRNAs in drosophilids. See caption of Fig. 5 for details. *Drosophila simulans:* Dsim; *Drosophila sechellia:* Dsec; *Drosophila melanogaster:* Dmel; *Drosophila yakuba:* Dyak; *Drosophila erecta:* Dere; *Drosophila ananassae:* Dana; *Drosophila pseudoobscura:* Dpse; *Drosophila persimilis:* Dper; *Drosophila willistoni:* Dwil; *Drosophila mojavensis:* Dmoj; *Drosophila virilis:* Dvir; *Drosophila grimshawi:* Dgri

As in the case of primate tRNAs, a substantial fraction of tRNAs can be traced back to the drosophilid ancestor and has been syntenically conserved since then, see Fig. 8. The seeding of new loci that subsequently are conserved in most species is again an ongoing process, accompanied by a relatively small rate of losses. As in the case of primates, the overwhelming part of the turnover is lineage specific and involves nearly half of the extant tRNA complement.

Due to the different genome version used in [20] and the UCSC multiz alignments only about 90 % of the tRNA genes can be related unambiguously between the two data set (see Methods for details). For the total of 2196 tRNAs, we identified 796 pairwise orthology relations with the multiz-anchored approach. The orthology map of [20] restricted to the same tRNAs comprises 5493 edges, 644 of which coincide with our much more restrictive orthology assignments. When clusters are joined, we increase the number of co-orthologs, thus increasing the number of ortholog pairs to 1808 of which 1061 coincide with the 1:1 assignments of [20]. Since the blast-regions used in [20] often correspond to very distant anchors, their orthology assignments are much more inclusive.

### Remolding of tRNAs

**Fig. 9:**
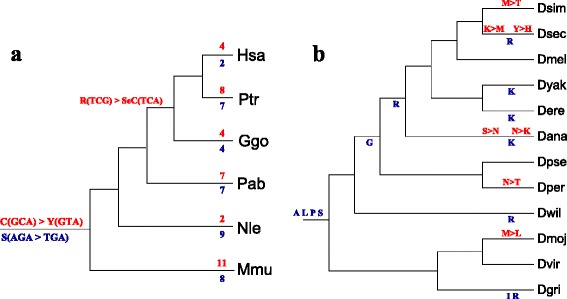
**a** Remolding events in primates (summary statistics only) and **b** drosophilids (affected isoacceptor classes). Isoacceptor remoldings are shown in *dark blue*, alloacceptor remoldings are given in *red*. Details of all anticodon changes are given in the Additional file 2

Numerous remolding events summarized in Table 1 and Fig. 9 were detected in both primates and drosophilids. A complete list of events is provided in Additional file 2. The remolding events identified here are largely congruent with those reported in [20] for fruit flies and [22] in primates. Our method is somewhat more sensitive and predicts significantly more tRNA remolding events. The overwhelming majority of events maps to the terminal branches of the phylogenetic trees. This concerns in particular almost all alloacceptor remoldings. Most likely, most or all of these “terminal” remolding events lead to non-functional tRNAs and already constitute pseudogenes. Despite the much greater phylogenetic depth of the drosophilid clade [60], we observe fewer remolding events. This may be explained at least in part by the larger total number of tRNAs in the primate genomes. Most of the detected remolding events occur close to the leaves of the phylogenetic tree. In principle they might be artefacts deriving from sequencing errors. While we cannot strictly rule out this interpretation, we deem it unlikely. First, the observed number of events would be unusually high: for primates we observe in total 73 remolding events at the leaves of the tree, in a sample of 1985 tRNAs. Within the three anticodon positions this would amount to a sequencing error rate of about 0.012, compared to an expected error rate in assembled contigs of ≪10^−4^/*nt* [61]. For drosophilids we observed 14 remoldings in 3348 tRNAs, amounting to a substitution rate of ≈0.0014. This also cannot be explained by sequencing errors. Second, we recover between 85 % and 95 % of the results in [20, 22] and detected additional putative remolding events although in part different genome assemblies were used. A much more plausible explanation therefore is that most remoldings affect tRNA function so that remolded tRNAs are unlikely to survive longterm and are rapidly pseudogenized and removed from the genome.

**Table 1:** Summary of remolding events in primates and drosophilids and comparison with previous studies [20, 22]

Primates			
	Common	[22] only	Novel
iso	9	0	9
allo	17	3	17
Drosophilids			
b)	Common	[20] only	Novel
iso	7	1	5
allo	4	1	3

The first column give the overlap between our data and a previous study

**Fig. 10:**
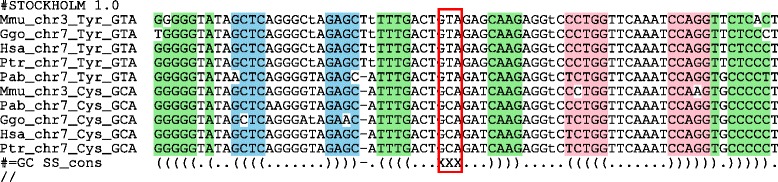
Alignment and secondary structure of tRNAs deriving from the Cys(GCA) →Tyr(GTA) remolding event predating the last common ancestor of human and rhesus. Descendants of both tRNAs have survived in all investigated genomes except *Nomascus*. The secondary structure is the standard tRNA structure

Interestingly, a small set of remoldings of threonine tRNAs was observed multiple times in primates, namely Thr: AGT →CGT, TGT. Surprisingly, we identified one alloacceptor remolding whose descendants persisted in primate genome since the common ancestor of human and rhesus. An ancestral tRNA Cys(GCA) gave rise to a remolded tRNA Tyr(GTA) whose sequence is still nearly identical to the Cys-decoding ancestor, see Fig. 10. While we have no direct evidence that this tRNA Tyr(GTA) is a functional tRNA, its evolutionary conservation is at least suggestive of some functional role.

### tRNA introns

Some tRNAs contain short introns. These are removed by a dedicated enzymatic machinery is not only fundamentally different from spliceosomal splicing but also that differs between Archaea or Eukaryotes and [62]. Nevertheless, most tRNA introns are located in the “canonical position”, one nucleotide 3’ to the anticodon [63]. We use tRNAs as an independent test for orthology assignment. We expect that either all or none of the members of a groups of (co-)orthologous tRNAs have an intron. This is indeed the case: In primates, there are 87 clusters of predicted orthologs in which all members carry an intron. In all other clusters none of the tRNAs has an intron. In drosophilids we found 49 clusters containing tRNAs with introns. All but a single one comprise tRNAs with introns only. The single exception is a tRNA Leu(CAA) cluster that also include single tRNA Leu(CAG) from the highly divergent *D. grimshawi*. It remains unclear whether this case constitutes a true change in intron structure, or whether the *D. grimshawi* tRNA is a false positive ortholog assignment. Despite a possible concerted evolution effect we observe that tRNA introns typically exhibit multiple substitutions and some insertions and deletions. In a small number of clusters of orthologous tRNAs in drosophilids we observe a considerably variation in intron length; in the extreme case introns have lengths between 21 and 52 nucleotides. This may not be unusual given that the phylogenetic depth of the drosophilids exceeds that of the mammalian radiation [60].

## Conclusions

Gene families that are subject to mechanisms of concerted evolution cannot be studied with traditional phylogenetic methods because concerted evolution rapidly erases all information about their evolutionary relationships from the sequences of paralogs. In this contribution we have investigated how synteny information can be harnessed in a systematic manner for this purpose. We have demonstrated that synteny *in principle* provides the necessary information as long as syntenically conserved sequence blocks are long enough to contain unique sequences that can be used as anchors. While it may seem desirable to use full-fledged sequence-based model such as OrthoAlign [43] to track genome evolution is full detail, such approaches do not scale to genome-wide surveys because of the computational efforts required. We reason that a stepwise workflow that first localizes the problem to individual gene clusters is a good compromise. These still can be prohibitively large, in particular in mammalian genomes. We therefore opted for a strategy that uses synteny information as much possible.

A useful outcome of the present study is to highlight the technical problems and difficulties associated with an accurate and quantitative analysis of the evolution of multicopy genes. Not surprisingly, the quality of the available data sources plays a critical role. While the annotation of tRNA gene and pseudogenes does not seem to pose much of a problem, there are several issues limiting the genome-wide multiple sequence alignments. On the one hand, coverage of alignable sequences can be a problem. In addition, as phylogenetic distances increase, the fraction of aligned DNA decreases, hence anchors will become sparser, making the synteny approach less accurate. An even more pressing problem, on the other hand, is the question whether aligned sequence blocks are really unique and thus are suitable as anchors. The differences in the results obtained with different anchor types indicate that genome-wide alignments provide less than perfect synteny anchors. Several factors seem to contribute. Most importantly, currently available MSA pipelines do not explicitly filter for unique sequences before computing alignment chains [57]. Highly conserved paralogous sequences therefore may lead to spurious anchors, which in turn may lead to false correspondences between homologous tRNAs. The concept of uniquely mappable sequence intervals [64, 65], originally developed for HTS data analysis, probably could be adapted to the construction of genomics MSAs that provide significantly more accurate anchor sets. This issue of ambiguities in MSAs needs to be addressed in future work as the development of new genome-wide alignment pipelines goes far beyond the scope of this work.

A surprising observation is that a large part of the inferred gain and loss of tRNAs is species specific. While this observation may be partially confounded by residual noise in the synteny assignments, it can be explained by a rapid copying of tRNAs followed by rapid pseudogenization. The tRNA model implemented in tRNAscan-SE [66] is very specific and distinguishes very stringently between tRNAs and tRNA pseudogenes that may differ by only a few point mutations from their functional ancestors. Reconstruction gain and loss events are largely consistent between the three levels of stringency in the definition of synteny, with most of the differences concentrated to the species-specific gains and losses.

Remolding events are observed predominantly at very shallow phylogenetic depths, indicating that most of them occur in pseudogenes. In contrast, remoldings that persist over large phylogenetic distances are rare and almost never change the isoacceptor class. Only a single deep alloacceptor remolding was observed. While it is unlikely that the remolded tRNA is functional in translation, it is well conceivable that the gene serves one of the recently described secondary function of a tRNA, as a source for miRNA-like small RNAs [67, 68], as sponge [69], or as a genomic insulator element influencing chromatin organization [70].

The workflow developed here is applicable not only to tRNAs (the focus of the current study) but also to other gene families evolving under concerted evolution or birth-death evolution showing patterns of rapid duplications and losses. This includes several families of non-conding RNAs but also rapidly evolving gene families such as olfactory receptors or the family of KRAB-ZNF in primates genes [9, 71]. This suggests that the development of a fully automatized pipeline capable of pulling together annotation and synteny information from diverse sources will be a worth-while endeavour.

## Methods

**Data sources.** A complete list of the genome sequences used in the study is given as Additional file 3. Transfer RNA genes were annotated with tRNAscan-SE [66] using the default model for eukaryotes.

For the MSA block based approach, we used the 16 primate multiz alignment [72] and the 26 insect multiz alignments [73] downloadable through the UCSC genome browser. In particular, these include the following assemblies of primate genomes: Human (*Homo sapiens*, hg19) and the primate genomes of *Gorilla gorilla* (gorGor3), *Macaca mulatta* (rheMac3), *Pan troglodytes* (panTro4) and *Pongo abelii* (ponAbe2). Pairwise human-primate alignments chains were also retrieved from UCSC genome browser. The complete set of protein-coding genes orthologous between human and the other primate species was retrieved from the Ensembl Compara data source (version 74) using the pipeline described in [74], which in turn is based on TreeBeST, see [75]. Where genome versions were different, we used blast to identify the exact coordinates in the UCSC primate genome.

**Cleaning of the raw **multiz** alignments** The multiz pipeline allows the same sequence to appear in more than one alignment block. This is the case in particular for duplicated genome regions. In order to remove all such ambiguities, we filtered the set of alignment blocks in the following manner: MAF blocks were first converted to a sorted BED format, describing position of each alignment block within a corresponding genome. Then for each tRNA, 5’ and 3’ adjacent alignment blocks without overlaps with any tRNA or other MAF block were identified.

**Orthologous proteins as synteny anchors** Denote by $\mathcal {P}_{ab}^{*}$ the set of all protein-coding genes annotated as orthologous pair between the species *a* and *b*. In order to resolve ambiguities between many-to-many co-orthologs we also use the chains for the alignment of *a* and *b*. A chain [76] specifies that the sequence intervals (*r*^*a*^,*s*^*a*^) and (*r*^*b*^,*s*^*b*^) are aligned. We retained only those orthologous pairs $p^{a},p^{b}\in \mathcal {P}_{ab}^{*}$ if there is an alignment chains so that *r*^*a*^≺*p*^*a*^≺*s*^*a*^ and *r*^*b*^≺*p*^*b*^≺*s*^*b*^. Denote the resulting set of 1:1 orthologs by $\mathcal {P}_{ab}$. For each tRNA *t*^*a*^ we consider the 10 proteins closest to *t*^*a*^ in the ortholog set. The coordinates of these proteins establish synteny anchors ${r_{i}^{a}},{r_{i}^{b}}$ and ${s_{i}^{a}},{s_{i}^{b}}$ for ${t_{i}^{a}}$ into species *b*. Using these anchors we compute the expected position ${t_{i}^{a}}(b)$ in genome *b* of an 1:1 ortholog of the tRNA ${t_{i}^{a}}$ by means of Eq.(1). We used the implementation of the coordinate lift-over described in [77]. For each ${t_{i}^{a}}$ both the coordinates of its 5’- and the 3’-end was used to specify its position. We finally choose the tRNA ${t_{j}^{b}}$ in *b* that is closest to the predicted position ${t_{i}^{a}}(b)$. First, we used *a*=human as references. To identify ortholog groups of tRNA that are lost in human we used in a second step all other primates as reference. Pairs of synteny candidates we retained only if they were identified reciprocally, i.e., with both *a* and *b* as reference. We omitted tRNAs for which suitable anchors could be identified and those for which no unambiguous coordinates could be computed in the other genomes. Consecutive pairs of orthologous tRNAs between the same anchor sets are compiled into genomic clusters. More details on the protein-anchors tRNA clusters are compiled in Additional file 4.

**Generalized list alignments** are based on an extension of the well-known Needleman-Wunsch alignment algorithm [37]. In addition to regular insertions, deletions and 1:1 (mis)matches, we also allow 1:*q* and *q*:1 mismatches to accommodate duplications, as shown by the following recursions of the modified Needleman-Wunsch alignment. As the algorithm is a dynamic programming algorithm, the complete solution is composed of smaller subsolutions. In this way the algorithm can be called recursively on on each subproblem.

2 $$ D_{ij} = \min \left\{ \begin{array}{ll} D_{i-1,j-1} + \delta({t^{a}_{i}},{t^{b}_{j}}) & \mathrm{M} \\ D_{i-1,j} + \delta({t^{a}_{i}}, -) & \mathrm{I}(a)\\ D_{i,j-1} + \delta(-,{t^{b}_{j}}) & \mathrm{I}(b)\\ D_{i-1,j-2} + \lambda\left({t^{a}_{i}};t^{b}_{j-1},{t^{b}_{j}}\right) & \mathrm{D}(a) \\ D_{i-2,j-1} + \lambda\left(t^{a}_{i-1},{t^{a}_{i}},{t^{b}_{j}}\right) & \mathrm{D}(b) \\ \end{array} \right.  $$

with the usual initialization score *D*_00_=0 for the empty alignment; The values $D_{i0}=\sum _{i'\le i}\delta (t^{a}_{i'},-)$ and $D_{0j}=\sum _{j'\le i}\delta (-,t^{b}_{j'})$ correspond to the insertion of prefixes in one of the sequences. Here M, I(*a*), I(*b*), D(*a*), and D(*b*) refer to (mis)matches between *a* and *b*, insertions in *a* and *b*, and duplications in *a* and *b*, respectively. Since the scoring in list alignments is dominated by excluding significantly different items from matching at all, we settled for a simple scoring model of the form

3 $$ \lambda({t^{a}_{i}},t^{b}_{j-1},{t^{b}_{j}}) = \delta({t^{a}_{i}},t^{b}_{j-1}) + \delta({t^{a}_{i}},{t^{b}_{j}}) + \eta  $$

for 1:2 matches, where *η*>0 is an extra penalty for the duplication and the two copies are otherwise scored independently like substitutions. 1:*q* matches for *q*>2 are treated analogously. For two tRNAs we use the dissimilarity score *δ*(*t*^′^,*t*^″^)=20, if Hamming distance between *t*^′^ and *t*^″^ is below a threshold value, and *δ*(*t*^′^,*t*^″^)=*∞* for more different tRNAs. Whereas 20 is a fixed value for the first scoring, later $\frac {\delta (t',t'')}{length(tRNA)} = 0.9$ was used such that the differences between two tRNAs are 10 % of their length. Since tRNA sequences have similar length overall the results are robust against such changes in the scoring function.

**Missing data in the synteny map** Although the construction of synteny map is rather conceptually simple, practical issues arise from less than perfect genome assemblies. tRNA genes that could not be placed in an unambiguous genomic context because no anchor or only a one-sided anchor was available were excluded from the analysis of tRNA clusters. This concerned 535 tRNAs (7 %) for drosophilids and 376 tRNAs (5 %) for the primate data. These tRNAs were included in detecting remolding events since the analysis was mainly based on alignments.

**Cograph Editing** Since the deviations from cograph structure is small in all cases, we used the brute force method described in [78].

**Comparison of different orthology reconstructions** Different estimates of orthology relations on the same set of genes yield different graph representations. We compare these in terms of the symmetric differences of their edge sets.

**Comparison with previous publications** A major difficulty in comparing our data with previous work from other groups is that tRNAs cannot be mapped to each other when different genome versions are used. Correspondences are listed in Additional file 5 for primates and in Additional file 6 for flies. In comparison with [20], a comparison of the coordinates systems was not possible for *D. willistoni*, *D. sechellia*, and *D. persimilis*. In the remaining species we were able to establish 1:2 correspondences for 2196 tRNAs. In the case where tRNAs could not be matched, 1:1 lift-over and sequence similarity were used to identify the most likely corresponding tRNA sequences. The remaining 216 tRNAs in the supplement of [20] could not be unambiguously assigned to 246 tRNAs appearing in our tRNA data.

## Abbreviations

APX hard, approximable hard; HTS, high-throughput sequencing; KRAB-ZNF, Küppel associated box zinc finger proteins; MAF, multiple [sequence] alignment [file] format; miRNA, microRNA ; MSA, multiple sequence alignment; NP hard, non-deterministic polynomial-time hard RNA, ribonucleic acid ; SINE, short interspersed nuclear element; snoRNA, small nucleolar RNA; tRNA, transfer RNA

## Additional files

Additional file 1 Quantitative comparison of (co)orthology assignments. Summary of used tRNAs and ortholog edges in primates and drosophilids and comparison of the different studies. (PDF 76 kb)

Additional file 2 tRNA remolding events. Details of detected remolding events including a comparison with already published data. (100 KB PDF)

Additional file 3 Genome versions used in the analysis. List of primate and drosophilid genomes and their versions. (PDF 75 kb)

Additional file 4 Additional Analyses of protein-anchored tRNA clusters. Additional figure showing distribution of tRNAs in primate species based on the orthologous protein anchor approach. (PDF 79 kb)

Additional file 5 List of primate tRNAs. Tab-separated table including positions and sequences of the tRNAs and translations to other genome versions. These tRNAs are used for the comparisons of the MSA block based approach and the orthologous proteins (OP) approach. (TXT 217 kb)

Additional file 6 List of drosophilid tRNAs. Tab-separated table including positions and sequences of the tRNAs and translations to other genome versions and FlyBase IDs. This list is used in the comparison of the MSA block based approach and the work of [20]. (TXT 305 kb)

## References

[1] Capra JA, Stolzer M, Durand D, Pollard KS (2013). How old is my gene?. Trends Genet.

[2] Holland PW (2013). Evolution of homeobox genes. Wiley Interdiscip Rev Dev Biol.

[3] Hiller M, Schaar BT, Indjeian VB, Kingsley DM, Hagey LR, Bejerano G (2012). A “forward genomics” approach links genotype to phenotype using independent phenotypic losses among related species. Cell Rep..

[4] Fitch WM (1970). Distinguishing homologous from analogous proteins. Syst Biol..

[5] Tatusov RL, Koonin EV, Lipman DJ (1997). A genomic perspective on protein families. Science.

[6] Altenhoff AM, Dessimoz C (2009). Phylogenetic and functional assessment of orthologs inference projects and methods. PLoS Comput Biol..

[7] Kristensen DM, Wolf YI, Mushegian AR, Koonin EV (2011). Computational methods for gene orthology inference. Brief Bioinf..

[8] Dalquen DA, Altenhoff AM, Gonnet GH, Dessimoz C (2013). The impact of gene duplication, insertion, deletion, lateral gene transfer and sequencing error on orthology inference: A simulation study. PLoS ONE.

[9] Nei M, Rooney AP (2005). Concerted and birth-and-death evolution of multigene families. Annu Rev Genet.

[10] Liao D (1999). Concerted evolution: Molecular mechanisms and biological implications. Am J Hum Genet..

[11] Amstutz H, Munz P, Heyer WD, Leupoid U, Kohli J (1985). Concerted evolution of tRNA genes: intergenic conversion among three unlinked serine tRNA genes in *S. pombe*. Cell.

[12] Teshima KM, Innan H (2004). The effect of gene conversion on the divergence between duplicated genes. Genetics.

[13] Chen JM, Cooper DN, Chuzhanova N, Férec C, Patrinos GP (2007). Gene conversion: mechanisms, evolution and human disease. Nat Rev Genet.

[14] Naidoo K, Steenkamp E, Coetzee MP, Wingfield MJ, Wingfield BD (2013). Concerted evolution in the ribosomal RNA cistron. PLoS One.

[15] Scienski K, Fay JCF, Conant GC (2015). Patterns of gene conversion in duplicated yeast histones suggest strong selection on a coadapted macromolecular complex. Genome Biol Evol.

[16] Weber MJ (2006). Mammalian small nucleolar RNAs are mobile genetic elements. PLoS Genet.

[17] Kramerov DA, Vassetzky NS (2011). Origin and evolution of SINEs in eukaryotic genomes. Heredity.

[18] Bermúdez-Santana C, Stephan-Otto Attolini C, Kirsten T, Engelhardt J, Prohaska SJ, Steigele S, Stadler PF (2010). Genomic organization of eukaryotic tRNAs. BMC Genomics.

[19] Michaud M, Cognar V, Duchêne AM, Maréchal-Drouard L (2011). A global picture of tRNA genes in plant genomes. Plant J..

[20] Rogers HH, Bergman CM, Griffiths-Jones S (2010). The evolution of tRNA genes in *Drosophila*. Genome Biol Evol.

[21] Wang PP, Ruvinsky I (2012). Family size and turnover rates among several classes of small non-protein-coding RNA genes in *Caenorhabditis* nematodes. Genome Biol Evol.

[22] Rogers HH, Griffiths-Jones S (2014). tRNA anticodon shifts in eukaryotic genomes. RNA.

[23] Hertel J, Stadler PF (2015). The expansion of animal microRNA families revisited. Life.

[24] Cantatore P, Gadaleta MN, Roberti M, Saccone C, Wilson AC (1987). Duplication and remoulding of tRNA genes during the evolutionary rearrangement of mitochondrial genomes. Nature.

[25] Rawlings TA, Collins TM, Bieler R (2003). Changing identities: tRNA duplication and remolding within animal mitochondrial genomes. Proc Nat Acad Sci USA.

[26] Sahyoun AH, Hölzer M, Jühling F, Höner zu Siederdissen C, Al-Arab M, Tout K, Marz M, Middendorf M, Stadler PF, Bernt M (2015). Towards a comprehensive picture of alloacceptor tRNA remolding in metazoan mitochondrial genomes. Nucleic Acids Res..

[27] Yona AH, Bloom-Ackermann Z, Frumkin I, Hanson-Smith V, Charpak-Amikam Y, Feng Q, Boeke JD, Dahan O, Pilpel Y (2013). tRNA genes rapidly change in evolution to meet novel translational demands. ELife.

[28] Giegé R, Sissler M, Florentz C (1998). Universal rules and idiosyncratic features in tRNA identity. Nucleic Acids Res.

[29] Elemento O, Gascuel O (2005). An exact and polynomial distance-based algorithm to reconstruct single copy tandem duplication trees. J Discr Algo..

[30] Zhang Y, Song G, Hsu CH, Miller W (2009). Simultaneous history reconstruction for complex gene clusters in multiple species. Pac Symp Biocomput.

[31] Vinař T, Brejová B, Song G, Siepel A (2010). Reconstructing histories of complex gene clusters on a phylogeny. J Comput Biol.

[32] Lajoie M, Bertrand D, El-Mabrouk N (2010). Inferring the evolutionary history of gene clusters from phylogenetic and gene order data. Mol Biol Evol.

[33] Tremblay Savard O, Bertrand D, El-Mabrouk N (2011). Evolution of orthologous tandemly arrayed gene clusters. BMC Bioinformatics.

[34] Brejová B, Kravec M, Landau GM, Vinař T (2014). Fast computation of a string duplication history under no-breakpoint-reuse. Philos Trans A Math Phys Eng Sci.

[35] Hellmuth M, Hernandez-Rosales M, Huber KT, Moulton V, Stadler PF, Wieseke N (2013). Orthology relations, symbolic ultrametrics, and cographs. J Math Biol..

[36] Hellmuth M, Wieseke N, Lechner M, Lenhof HP, Middendorf M, Stadler PF (2015). Phylogenetics from paralogs. Proc Nat Acad Sci USA.

[37] Needleman SB, Wunsch CD (1970). A general method applicable to the search for similarities in the amino acid sequence of two proteins. J Mol Biol.

[38] Kondrak G (2000). A new algorithm for the alignment of phonetic sequences. Proceedings of NAACL 2000 1st Meeting of the North American Chapter of the Association for Computational Linguistics.

[39] Eger S (2013). Sequence alignment with arbitrary steps and further generalizations, with applications to alignments in linguistics. Information Sci..

[40] Fried C, Hordijk W, Prohaska SJ, Stadler CR, Stadler PF (2004). The footprint sorting problem. J Chem Inf Comput Sci..

[41] Dondi R, El-Mabrouk N (2013). Aligning and Labeling Genomes Under the Duplication-Loss Model.

[42] Benzaid B, Dondi R, El-Mabrouk N. Duplication-loss genome alignment: Complexity and algorithm In: Dediu CA-H, Martin-Vide, Truthe B, editors. Language and Automata Theory and Applications, (LATA). Berlin, Heidelberg: Springer: 2013. p. 116–27.

[43] Tremblay-Savard O, Benzaid B, Lang BF, El-Mabrouk N (2015). Evolution of tRNA repertoires in Bacillus inferred with OrthoAlign. Mol Biol Evol.

[44] Bernt M, Merkle D, Rasch K, Fritzsch G, Perseke M, Bernhard D, Schlegel M, Stadler PF, Middendorf M (2007). CREx: Inferring genomic rearrangements based on common intervals. Bioinformatics.

[45] Hu F, Lin Y, Tang J (2014). MLGO: phylogeny reconstruction and ancestral inference from gene-order data. BMC Bioinformatics.

[46] Feijão P (2015). Reconstruction of ancestral gene orders using intermediate genomes. BMC Bioinformatics.

[47] Braga MDV, Stoye J (2015). Sorting linear genomes with rearrangements and indels. IEEE/ACM Trans Comp Biol Bioinf..

[48] Prohaska S, Fried C, Flamm C, Wagner G, Stadler PF (2004). Surveying phylogenetic footprints in large gene clusters: Applications to Hox cluster duplications. Mol Phyl Evol..

[49] Otto W, Stadler PF, Prohaska SJ, Giancarlo R, Manzini G (2011). Phylogenetic footprinting and consistent sets of local aligments. CPM 2011. Lecture Notes in Computer Science, vol. 6661.

[50] Hernandez-Rosales M, Hellmuth M, Wieseke N, Huber KT, Moulton V, Stadler PF (2012). From event-labeled gene trees to species trees. BMC Bioinformatics.

[51] Lafond M, Semeria M, Swenson KM, Tannier E, El-Mabrouk N (2013). Gene tree correction guided by orthology. BMC Bioinformatics.

[52] Lafond M, El-Mabrouk N (2014). Orthology and paralogy constraints: satisfiability and consistency. BMC Genomics.

[53] Lafond M, Dondi R, El-Mabrouk N (2016). The link between orthology relations and gene trees: a correction perspective. Alg Mol Biol..

[54] Corneil DG, Lerchs H, Steward Burlingham L (1981). Complement reducible graphs. Discr Appl Math..

[55] Liu Y, Wang J, Guo J, Chen J (2012). Complexity and parameterized algorithms for cograph editing. Theor Comp Sci..

[56] Farris JS (1977). Phylogenetic analysis under Dollo’s law. Syst Zool.

[57] Blanchette M, Kent WJ, Riemer C, Elnitski L, Smit AFA, Roskin KM, Baertsch R, Rosenbloom K, Clawson H, Green ED, Haussler D, Miller W (2004). Aligning multiple genomic sequences with the threaded blockset aligner. Genome Res..

[58] Lokshtanov D, Mancini F, Papadopoulos C (2010). Characterizing and computing minimal cograph completions. Discrete Appl Math..

[59] Drosophila 12 Genomes Consortium: Evolution of genes and genomes on the *Drosophila* phylogeny. Nature. 2007; 450:203–18. doi:10.1038/nature06341. 10.1038/nature06341 17994087

[60] Drosophila 12 Genomes Consortium: Evolution of genes and genomes on the *Drosophila* phylogeny. Nature. 2007; 450:203–18. doi:10.1038/nature06341. 10.1038/nature06341 17994087

[61] Powers JG, Weigman VJ, Shu J, Pufky JM, Cox D, Hurban P (2013). Efficient and accurate whole genome assembly and methylome profiling of *E. coli*. BMC Genomics.

[62] Lopes RR, Kessler AC, Polycarpo C, Alfonzo JD (2015). Cutting, dicing, healing and sealing: the molecular surgery of tRNA. Wiley Interdiscip Rev RNA.

[63] Yoshihisa T (2014). Handling tRNA introns, archaeal way and eukaryotic way. Front Genet.

[64] Treangen TJ, Salzberg SL (2012). Repetitive DNA and next-generation sequencing: computational challenges and solutions. Nature Rev Gen..

[65] Storvall H, Ramsköld D, Sandberg R (2013). Efficient and comprehensive representation of uniqueness for next-generation sequencing by minimum unique length analyses. PLoS ONE.

[66] Lowe TM, Eddy SR (1997). tRNAscan-SE: A program for improved detection of transfer RNA genes in genomic sequence. Nucleic Acids Res..

[67] Lee YS, Shibata Y, Malhotra A, Dutta A (2009). A novel class of small RNAs: tRNA-derived RNA fragments (tRFs). Genes Dev..

[68] Kumar P, Anaya J, Mudunuri SB, Dutta A (2014). Meta-analysis of tRNA derived RNA fragments reveals that they are evolutionarily conserved and associate with AGO proteins to recognize specific RNA targets. BMC Biol.

[69] Lalaouna D, Carrier MC, Semsey S, Brouard JS, Wang J, Wade JT, Massé E (2015). A 3’ external transcribed spacer in a tRNA transcript acts as a sponge for small RNAs to prevent transcriptional noise. Mol Cell.

[70] Van Bortle K, Corces VG (2012). tDNA insulators and the emerging role of TFIIIC in genome organization. Transcription.

[71] Eirín-López JM, Rebordinos L, Rooney AP, Rozas J (2012). The birth- and- death evolution of multigene families revisited. Genome Dyn..

[72] UCSC Genome Browser: Multiple alignments of 19 mammalian (16 primate) genomes with Human. 2013. http://hgdownload.cse.ucsc.edu/goldenPath/hg38/multiz20way/maf/. Accessed 14 Oct 2015.

[73] UCSC Genome Browser: Multiple alignments of 26 insects with D. melanogaster. 2014. http://hgdownload.cse.ucsc.edu/goldenPath/dm6/multiz27way/maf/. Accessed 14 Oct 2015.

[74] Ensembl. Ensembl: Collecting Orthologs. 2012. http://www.geocities.jp/ancientfishtree/EnsOrthoCollection.html.

[75] Vilella AJ, Severin J, Ureta-Vidal A, Heng L, Durbin R, Birney E (2009). EnsemblCompara GeneTrees: Complete, duplication-aware phylogenetic trees in vertebrates. Genome Res..

[76] Kent WJ, Baertsch R, Hinrichs A, Miller W, Haussler D (2003). Evolution’s cauldron: duplication, deletion, and rearrangement in the mouse and human genomes. Proc Natl Acad Sci USA.

[77] Arnold C. Translating Coordinates Between Species. Univ. Leipzig: Univ. Leipzig. Manual and Software; 2014.

[78] Berkemer SJ. Cograph Editing: An Approach to Adjust the Orthology Relation for the Reconstruction of Phylogenetic Trees. 2012. http://www.bioinf.uni-leipzig.de/~bsarah/cograph_editing.zip. BSc thesis.

