# Supplementary material for: Orthologs, turn-over, and remolding of tRNAs in primates and fruit flies
Source: BMC Genomics. 2016 Aug 11;17:617. doi: 10.1186/s12864-016-2927-4 (PMC4981973; doi:10.1186/s12864-016-2927-4)
Supplement: Additional file 1 — Quantitative comparison of (co)orthology assignments. Summary of used tRNAs and ortholog edges in primates and drosophilids and comparison of the different studies. (PDF 76 kb) [file 12864_2016_2927_MOESM1_ESM.pdf]

SUPPLEMENTAL MATERIAL

Orthologs, turn-over, and remolding of tRNAs in primates and fruit flies

Additional file 1

Cristian A Velandia-Huerto[1†], Sarah J Berkemer[2,3†], Anne Hoffmann[3], Nancy Retzlaff[2,3], Liliana Romero Marroquín[1], Maribel Hernández Rosales[4], Peter F Stadler[2,3,5,6,7,8*] and Clara I Bermúdez-Santana[1]

[*]Correspondence:
studla@bioinf.uni-leipzig.de
[3]Bioinformatics Group,
Department of Computer Science,
and Interdisciplinary Center for
Bioinformatics, Universität
Leipzig, Härtelstraße 16–18,
D-04107 Leipzig, Germany
Full list of author information is
available at the end of the article
[†]Equal contributor

	MSA	OP	overlap
H. sapiens	858	434	432
P. troglodytes	566	336	336
G. gorilla	549	345	345
P. abelii	661	303	303
N. leucogenys	551	NA	NA
M. mulatta	578	317	317
total	3763	1735	1733

Table 1 Table showing the numbers of primate tRNAs used in the approaches and the number of overlapping tRNAs.

	MSA	Rogers et al.'10	overlap
D. melanogaster	295	297	275
D. pseudoobscura	294	262	135
D. simulans	267	255	245
D. sechellia	309	294	NA
D. yakuba	375	317	300
D. erecta	284	283	278
D. ananassae	469	296	285
D. persimilis	298	258	NA
D. willistoni	457	234	NA
D. virilis	269	242	232
D. mojavensis	264	234	229
D. grimshawi	259	225	216
total	3840	3197	2196

Table 2 Table showing the numbers of tRNAs in drosophilids used in the approaches and the number of overlapping tRNAs.

MSA - joined clusters	1986
OP	4957
overlap of MSA and OP	1653

Table 3 Table showing the numbers of orthologous edges in primates in each approach and their overlap.

MSA - unjoined clusters	796
MSA - joined clusters	1808
Rogers et al. 2010	5493
overlap of MSA (unjoined) and Rogers et al. '10	644
overlap of MSA (joined) and Rogers et al. '10	1061

Table 4 Table showing the numbers of orthologous edges in drosophilids in each approach and their overlap.

Author details
[1]Biology Department, Universidad Nacional de Colombia, Carrera 45 # 26-85, Edif. Uriel Gutiérrez, Bogotá D.C, Colombia. [2] Max Planck Institute for Mathematics in the Sciences, Inselstraße 22, D-04103 Leipzig, Germany. [3]Bioinformatics Group, Department of Computer Science, and Interdisciplinary Center for Bioinformatics, Universität Leipzig, Härtelstraße 16–18, D-04107 Leipzig, Germany. [4]Instituto de Matemáticas, UNAM Juriquilla, Adolfo Villaseñor #12, Constituyentes del Parque, MX-76147 Santiago de Querétaro, QE, México. [5] Fraunhofer Institut for Cell Therapy and Immunology, Perlickstraße 1, D-04103 Leipzig, Germany. [6]Department of Theoretical Chemistry, University of Vienna Währinger Straße 17, A-1090 Vienna, Austria. [7] Center for non-coding RNA in Technology and Health, Grønegårdsvej 3, DK-1870 Frederiksberg C, Denmark. [8] Santa Fe Institute, 1399 Hyde Park Rd., NM87501 Santa Fe, USA.
