# Supplementary material for: Orthologs, turn-over, and remolding of tRNAs in primates and fruit flies
Source: BMC Genomics. 2016 Aug 11;17:617. doi: 10.1186/s12864-016-2927-4 (PMC4981973; doi:10.1186/s12864-016-2927-4)
Supplement: Additional file 2 — tRNA remolding events. Details of detected remolding events including a comparison with already published data. (100 KB PDF) [file 12864_2016_2927_MOESM2_ESM.pdf]

SUPPLEMENTAL MATERIAL

Orthologs, turn-over, and remolding of tRNAs in primates and fruit flies

Additional file 2

Cristian A Velandia-Huerto[1†], Sarah J Berkemer[2,3†], Anne Hoffmann[3], Nancy Retzlaff[2,3], Liliana Romero Marroquín[1], Maribel Hernández Rosales[4], Peter F Stadler[2,3,5,6,7,8*] and Clara I Bermúdez-Santana[1]

*Correspondence: studla@bioinf.uni-leipzig.de
[3]Bioinformatics Group, Department of Computer Science, and Interdisciplinary Center for Bioinformatics, Universität Leipzig, Härtelstraße 16–18, D-04107 Leipzig, Germany
Full list of author information is available at the end of the article
†Equal contributor

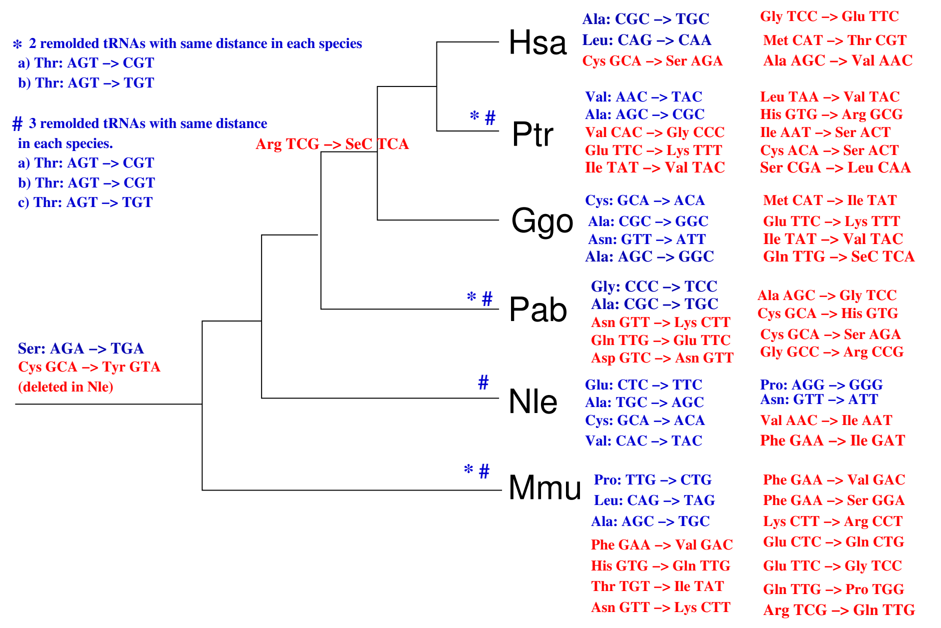

Figure 1 Remolding events of tRNAs in primates. Isoacceptor changes are marked in blue, alloacceptor changes in red. Species abbreviations: human, *Homo sapiens*: Hsa; chimapanzee, *Pan troglodytes*: Ptr; gorilla, *Gorilla gorilla*: Ggo; orangutan, *Pongo abelii*: Pab; gibbon, *Nomascus leucogenys*: Nle; rhesus macaque, *macaca mulatta*: Mmu.

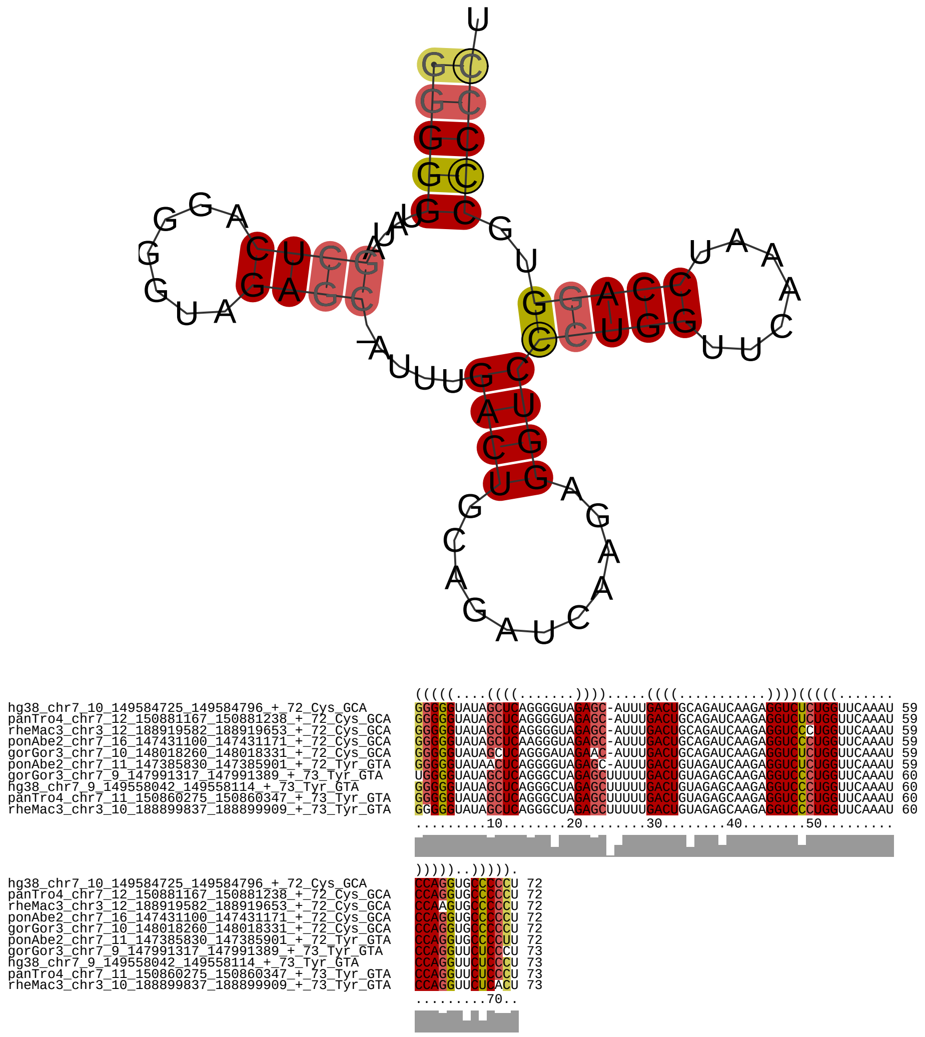

Figure 2 Secondary structure (top) and alignment (bottom) of primate tRNA sequences detected within an alloacceptor change remolding event close to the root of the phylogenetic tree. Species abbreviations: human, *Homo sapiens*: Hsa; chimapanzee, *Pan troglodytes*: Ptr; gorilla, *Gorilla gorilla*: Ggo; orangutan, *Pongo abelii*: Pab; gibbon, *Nomascus leucogenys*: Nle; rhesus macaque, *macaca mulatta*: Mmu.

	common	Ref.	our data
allo	Ser-AGA - Cys-GCA	Glu-CTC - Ala-TGC	Met-CAT - Thr-CGT
	Tyr-GTA - Cys-GCA	Arg-GCG - Cys-GCA	Met-CAT - Ile-TAT
	Arg-GCG - His-GTG	SeC-TCA - Cys-GCA	Ile-AAT - Ser-ACT
	Ser-ACT - Ile-AAT		Gln-TTG - Glu-TTC
	Val-TAC - Leu-TAA		Gln-CTG - Glu-CTC
	Leu-CAA - Ser-CGA		Asp-GTC - Asn-GTT
	Gln-TTG - Arg-TCG		Ala-AGC - Gly-TCC
	Thr-CGT - Met-CAT		Cys-GCA - His-GTG
	Glu-TTC - Gly-TCC		Val-AAC - Ile-AAT
	Lys-CTT - Asn-GTT		Phe-GAA - Val-GAC
	Val-CAC - Gly-CCC		His-GTG - Gln-TTG
	Glu-TTC - Lys-TTT		Thr-TGT - Ile-TAT
	Arg-CCG - Gly-CCC		Phe-GAA - Ser-GCA
	Ile-GAT - Phe-GAA		Lys-CTT - Arg-CCT
	Leu-CAA - Met-CAT		Gln-TTG - Pro-TGG
	Val-TAC - Ile-TAT		Gln-TTG - SeC-TCA
	Val-AAC - Ala-AGC		Arg-TCG - SeC-TCA
	common	Ref.	our data
iso	Leu-CAA - Leu-CAG		Leu-CAG - Leu-TAG
	Cys-ACA - Cys-GCA		Thr-AGT - Thr-CGT
	Glu-TTC - Glu-CTC		Thr-AGT - Thr-TGT
	Asn-ATT - Asn-GTT		Val-AAC - Val-TAC
	Ala-GGC - Ala-AGC		Val-CAC - Val-TAC
	Ala-TGC - Ala-CGC		Ala-AGC - Ala-CGC
	Thr-CGT - Thr-TGT		Ala-TGC - Ala-AGC
	Ser-TGA - Ser-AGA		Pro-AGG - Pro-GGG
	Gly-TCC - Gly-CCC		Pro-TTG - Pro-CTG

Table 1 List of remolding events detected in both primate datasets, just in Rogers et al and only in our data.

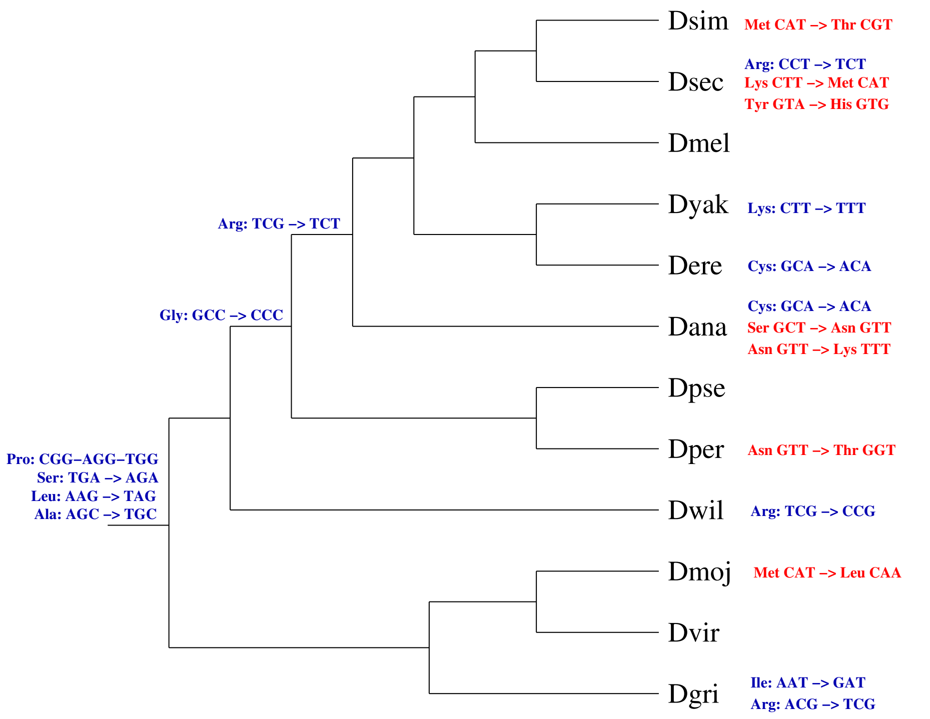

Figure 3 Remolding events of tRNAs in drosophilids. Isoacceptor changes are marked in blue, alloacceptor changes in red. *Drosophila simulans:* Dsim; *Drosophila sechellia:* Dsec; *Drosophila melanogaster:* Dmel; *Drosophila yakuba:* Dyak; *Drosophila erecta:* Dere; *Drosophila ananassae:* Dana; *Drosophila pseudoobscura:* Dpse; *Drosophila persimilis:* Dper; *Drosophila willistoni:* Dwil; *Drosophila mojavensis:* Dmoj; *Drosophila virilis:* Dvir; *Drosophila grimshawi:* Dgri.

	common	Ref.	our data
allo	Met-CAT - Thr-CGT<br>Lys-CTT - Met-CAT<br>Tyr-GTA - His-GTG<br>Asn-GTT - Lys-TTT	Asp-GTC - Asn-GTT	Ser-GCT - Asn-GTT<br>Asn-GTT - Thr-GGT<br>Met-CAT - Leu-CAA
	common	Ref.	our data
iso	Arg-CCT - Arg-TCT<br>Ser-TGA - Ser-AGA<br>Arg-TCG - Arg-TCT<br>Gly-GCC - Gly-CCC<br>Cys-GCA - Cys-ACA<br>Pro: AGG,CGG,TGG<br>Arg-ACG - Arg-TCG	Cys-GCA - Cys-ACA	Leu-AAG - Leu-TAG<br>Ala-AGC - Ala-TGC<br>Lys-CTT - Lys-TTT<br>Arg-TCG - Arg-CCG<br>Ile-AAT - Ile-GAT

Table 2 List of remolding events detected in both drosophilids datasets, just in Rogers et al and only in our data.

Author details
[1]Biology Department, Universidad Nacional de Colombia, Carrera 45 # 26-85, Edif. Uriel Gutiérrez, Bogotá D.C, Colombia. [2] Max Planck Institute for Mathematics in the Sciences, Inselstraße 22, D-04103 Leipzig, Germany. [3]Bioinformatics Group, Department of Computer Science, and Interdisciplinary Center for Bioinformatics, Universität Leipzig, Härtelstraße 16–18, D-04107 Leipzig, Germany. [4]Instituto de Matemáticas, UNAM Juriquilla, Adolfo Villaseñor #12, Constituyentes del Parque, MX-76147 Santiago de Querétaro, QE, México. [5] Fraunhofer Institut for Cell Therapy and Immunology, Perlickstraße 1, D-04103 Leipzig, Germany. [6]Department of Theoretical Chemistry, University of Vienna Währinger Straße 17, A-1090 Vienna, Austria. [7] Center for non-coding RNA in Technology and Health, Grønegårdsvej 3, DK-1870 Frederiksberg C, Denmark. [8] Santa Fe Institute, 1399 Hyde Park Rd., NM87501 Santa Fe, USA.
