# Supplementary material for: Orthologs, turn-over, and remolding of tRNAs in primates and fruit flies
Source: BMC Genomics. 2016 Aug 11;17:617. doi: 10.1186/s12864-016-2927-4 (PMC4981973; doi:10.1186/s12864-016-2927-4)
Supplement: Additional file 3 — Genome versions used in the analysis. List of primate and drosophilid genomes and their versions. (PDF 75 kb) [file 12864_2016_2927_MOESM3_ESM.pdf]

SUPPLEMENTAL MATERIAL

Orthologs, turn-over, and remolding of tRNAs in primates and fruit flies

Additional file 3

Cristian A Velandia-Huerto[1†], Sarah J Berkemer[2,3†], Anne Hoffmann[3], Nancy Retzlaff[2,3], Liliana Romero Marroquín[1], Maribel Hernández Rosales[4], Peter F Stadler[2,3,5,6,7,8*] and Clara I Bermúdez-Santana[1]

[*]Correspondence: studla@bioinf.uni-leipzig.de
[3]Bioinformatics Group, Department of Computer Science, and Interdisciplinary Center for Bioinformatics, Universität Leipzig, Härtelstraße 16–18, D-04107 Leipzig, Germany
Full list of author information is available at the end of the article
[†]Equal contributor

Primates	MSA block based approach	orthologous proteins approach
H. sapiens	Dec. 2013 (GRCh38hg38)	Feb. 2009 (GRCh37hg19)
P. troglodytes	Feb. 2011 (CSAC 2.1.4panTro4)	Feb. 2011 (CSAC 2.1.4panTro4)
M. mulatta	Oct. 2010 (BGI CR_1.0rheMac3)	Oct. 2010 (BGI CR_1.0rheMac3)
G. gorilla	May 2011 (gorGor3.1gorGor3)	May 2011 (gorGor3.1gorGor3)
N. leucogenys	Oct. 2012 (GGSC Nleu3.0nomLeu3)	-
P. abelii	Jul. 2007 (WUGSC 2.0.2ponAbe2)	Jul. 2007 (WUGSC 2.0.2ponAbe2)

Table 1 Table showing the genome versions used in the analysis done based on multiz and linear interpolation method.

Drosophilids	MSA block based approach
D. melanogaster	Aug. 2014 (BDGP Release 6 + ISO1 MTdm6)
D. pseudoobscura	Apr. 2013 (BCM Dpse_3.0droPse3)
D. simulans	Apr. 2005 (WUGSC 1.0droSim1)
D. sechellia	Oct. 2005 (BroaddroSec1)
D. yakuba	Jun. 2006 (Flybase dyak_caf1droYak3)
D. erecta	Feb. 2006 (Agencourt CAF1droEre2)
D. ananassae	Feb. 2006 (Agencourt CAF1droAna3)
D. persimilis	Oct. 2005 (BroaddroPer1)
D. willistoni	Aug. 2006 (JCVI dwil_caf1droWil2)
D. virilis	Feb. 2006 (Agencourt CAF1droVir3)
D. mojavensis	Feb. 2006 (Agencourt CAF1droMoj3)
D. grimshawi	Feb. 2006 (Agencourt CAF1droGri2)

Table 2 Table showing the genome versions used in the analysis.

Author details
[1]Biology Department, Universidad Nacional de Colombia, Carrera 45 # 26-85, Edif. Uriel Gutiérrez, Bogotá D.C, Colombia. [2] Max Planck Institute for Mathematics in the Sciences, Inselstraße 22, D-04103 Leipzig, Germany. [3]Bioinformatics Group, Department of Computer Science, and Interdisciplinary Center for Bioinformatics, Universität Leipzig, Härtelstraße 16–18, D-04107 Leipzig, Germany. [4]Instituto de Matemáticas, UNAM Juriquilla, Adolfo Villaseñor #12, Constituyentes del Parque, MX-76147 Santiago de Querétaro, QE, México. [5] Fraunhofer Institut for Cell Therapy and Immunology, Perlickstraße 1, D-04103 Leipzig, Germany. [6]Department of Theoretical Chemistry, University of Vienna Währinger Straße 17, A-1090 Vienna, Austria. [7] Center for non-coding RNA in Technology and Health, Grønegårdsvej 3, DK-1870 Frederiksberg C, Denmark. [8] Santa Fe Institute, 1399 Hyde Park Rd., NM87501 Santa Fe, USA.
