# Supplementary material for: Orthologs, turn-over, and remolding of tRNAs in primates and fruit flies
Source: BMC Genomics. 2016 Aug 11;17:617. doi: 10.1186/s12864-016-2927-4 (PMC4981973; doi:10.1186/s12864-016-2927-4)
Supplement: Additional file 4 — Additional Analyses of protein-anchored tRNA clusters. Additional figure showing distribution of tRNAs in primate species based on the orthologous protein anchor approach. (PDF 79 kb) [file 12864_2016_2927_MOESM4_ESM.pdf]

SUPPLEMENTAL MATERIAL

Orthologs, turn-over, and remolding of tRNAs in primates and fruit flies

Additional file 4

Cristian A Velandia-Huerto[1†], Sarah J Berkemer[2,3†], Anne Hoffmann[3], Nancy Retzlaff[2,3], Liliana Romero Marroquín[1], Maribel Hernández Rosales[4], Peter F Stadler[2,3,5,6,7,8*] and Clara I Bermúdez-Santana[1]

*Correspondence: studla@bioinf.uni-leipzig.de
[3]Bioinformatics Group, Department of Computer Science, and Interdisciplinary Center for Bioinformatics, Universität Leipzig, Härtelstraße 16–18, D-04107 Leipzig, Germany
Full list of author information is available at the end of the article
†Equal contributor

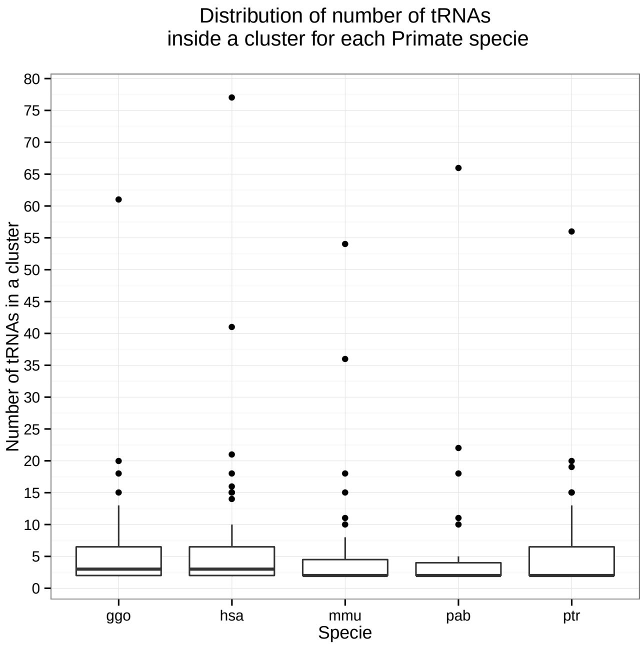

Figure 1 Distribution of number of tRNA elements inside clusters along primate species. Tags are: hsa (*H. sapiens*), ggo (*G. gorilla*), mmu (*M. mulatta*), ptr (*P. troglodytes*) and pab (*P. abelii*)

Author details
[1]Biology Department, Universidad Nacional de Colombia, Carrera 45 # 26-85, Edif. Uriel Gutiérrez, Bogotá D.C, Colombia. [2] Max Planck Institute for Mathematics in the Sciences, Inselstraße 22, D-04103 Leipzig, Germany. [3]Bioinformatics Group, Department of Computer Science, and Interdisciplinary Center for Bioinformatics, Universität Leipzig, Härtelstraße 16–18, D-04107 Leipzig, Germany. [4]Instituto de Matemáticas, UNAM Juriquilla, Adolfo Villaseñor #12, Constituyentes del Parque, MX-76147 Santiago de Querétaro, QE, México. [5] Fraunhofer Institut for Cell Therapy and Immunology, Perlickstraße 1, D-04103 Leipzig, Germany. [6]Department of Theoretical Chemistry, University of Vienna Währinger Straße 17, A-1090 Vienna, Austria. [7] Center for non-coding RNA in Technology and Health, Grønegårdsvej 3, DK-1870 Frederiksberg C, Denmark. [8] Santa Fe Institute, 1399 Hyde Park Rd., NM87501 Santa Fe, USA.
